# MirathQA: A dataset for evaluating large language models on Hanbali Islamic inheritance reasoning tasks

**DOI:** 10.1016/j.dib.2026.112589

**Published:** 2026-02-13

**Authors:** Ameera Almasoud, Sharefah Al-Ghamdi, Reem Alqifari, Noof Alfear, Hend Al-Khalifa

**Affiliations:** Department of Information Technology, College of Information and Computer Sciences, King Saud University, Riyadh, Saudi Arabia

**Keywords:** Islamic inheritance, Hanbali jurisprudence, Natural language processing, Large language models, Benchmark, Fiqh dataset, Arabic Language, Dataset annotation

## Abstract

Islamic inheritance (Muwārīth/مواريث) refers to the distribution of a deceased person's estate among qualified heirs in accordance with Sharia laws derived from the Qur’an and Sunnah. This dataset focuses specifically on the Hanbali school of jurisprudence (المذهب الحنبلي), one of the four major Sunni schools, which has distinct rulings on residuary heirs (taʿsīb/التعصيب) and blocking mechanisms (al-ḥajb/الحجب). Despite recent advances, Arabic large language models (LLMs) often struggle with tasks requiring exact arithmetic, multi-step reasoning, and strict adherence to domain-specific rules. These challenges are particularly evident in Islamic inheritance law (ʿilm al-farāʾiḍ/علم الفرائض), where case resolution demands sequential rule application, identification of heirs and blocked heirs (ḥijb/حجب), handling of proportional reduction (ʿawl/عول) and return (radd/رد), and accounting for juristic differences. The lack of well-structured Arabic datasets further restricts systematic evaluation in this area. To address this gap, we introduce the MirathQA, comprising over 1394 questions derived from 242 real-life inheritance cases collected from different Arabic sources. Each case includes a textual description, verified solution, heirs and their corresponding shares, and annotations for blocked or modified heirs. Additional multiple-choice and true/false questions assess reasoning skills such as exclusion and proportional reduction. The dataset is divided into training (70%), validation (15%), and test (15%) splits at the case level to prevent leakage and ensure reproducibility. It is also provided in both CSV and Excel formats. This resource establishes a benchmark for evaluating Arabic LLM reasoning, while also serving education, interdisciplinary research, and legal AI applications.

Specifications Table

**Table:** 

Subject	Computer Sciences
Specific subject area	Arabic NLP; Islamic Law; Dataset Evaluation
Type of data	Table; Text; Raw; Annotated; Processed
Data collection	Data were manually collected and systematically structured through extraction of jurisprudential Islamic inheritance cases from authoritative and publicly accessible Fiqh sources. These included the Top 100 Inheritance Problems collection from AlMwareeth, official Fiqh textbooks issued by the Saudi Ministry of Education and verified Islamic law repositories such as the Shamela Digital Library. Structured text parsing using custom Python scripts was applied, followed by manual verification. Inclusion focused on complete, well-defined cases covering diverse heir compositions, blocked scenarios, and adjusted shares. Data were standardized using consistent formatting. The dataset is publicly stored in the Mendeley Data repository.
Data source location	The dataset was extracted from the Top 100 Inheritance Problems collection from AlMwareeth, official Fiqh textbooks issued by the Saudi Ministry of Education and verified Islamic law repositories such as the Shamela Digital Library. The dataset is publicly stored in the Mendeley Data repository.
Data accessibility	Repository name: MirathQA DOI: 10.17632/7jhycpbdpw.4 Direct URL to data: https://data.mendeley.com/datasets/7jhycpbdpw/4 Instructions for accessing these data: The dataset is publicly available via the provided URL and can be accessed without registration or special permissions.
Related research article	None

## 1 Value of the Data

• Educational and Training Applications: The dataset facilitates the creation of AI-driven tutoring systems for Islamic inheritance law (ʿilm al-farāʾiḍ/علم الفرائض), providing incremental feedback and interactive learning experiences for students.
• Chain-of-Thought (CoT) and Legal Arithmetic Reasoning: The dataset supports the evaluation and improvement of multi-step legal arithmetic reasoning and chain-of-thought–based reasoning in large language models.
• Judicial and Decision-Support Systems: The dataset can be utilized to verify inheritance computations, identify inaccuracies in intricate cases (e.g., ʿAwl and Radd), and facilitate initial decision-making in Sharia-based legal frameworks.
• Comparative and Multilingual Legal NLP Research: While based on Hanbali jurisprudence, the dataset serves as a basis for comparative analyses among Islamic legal schools and facilitates multilingual legal NLP and cross-lingual transfer learning investigations.
• Rule-Based systems: The cases are structured in a way that makes it easier to study rule-based systems (like smart contracts for dividing up inheritances) and to do quantitative research on inheritance patterns, family structures, and socio-economic effects.

## 2 Background

Islamic inheritance law (ʿilm al-farāʾiḍ/علم الفرائض) constitutes a fundamental aspect of Islamic jurisprudence, governed by stringent mathematical and logical principles. It delineates the process of distributing a deceased individual's wealth among their legitimate heirs. It is founded on principles delineated in the Qur'an, Sunnah, and classical legal commentaries [1,2]. This area is very hard for automated reasoning systems since it is so complicated, especially when there are several heirs, residuary shares (taʾsīb/تعصيب), modified fractions (ʿawl/عول), and blocked heirs (ḥajb/حجب).

The system prescribes fixed shares for specific relatives such as spouses, children, parents, and siblings, while also accounting for more complex elements such as residuary heirs (taʾsīb/تعصيب), adjusted fractional shares (ʿawl/عول), and exclusionary rules (ḥajb/حجب) that can block certain heirs based on hierarchical relationships.

Unlike many secular legal systems, which often allow broad personal discretion in testamentary decisions, Islamic inheritance law permits only one-third of the estate to be distributed through a will. The remaining two-thirds must follow the strictly defined shares outlined in Islamic sources, ensuring justice, transparency, and the protection of vulnerable family members [3].

The system's intricacy makes it one of the most challenging areas for automated legal reasoning and computational implementation, particularly in scenarios involving multiple heirs and overlapping inheritance rules. Its depth and complexity reflect its moral and religious importance within Islam, with detailed prescriptions found notably in Surah An-Nisāʾ (verses 11 [4], 12 [5], and 176 [6]), making it one of the most elaborately codified aspects of Islamic law.

Large language models (LLMs) have demonstrated potential in legal and religious reasoning tasks in recent years [7]; nevertheless, there is a scarcity of datasets that evaluate these models in Arabic and authentic Islamic contexts. The MirathQA dataset aims to address this deficiency by offering a high-quality, annotated compilation of authentic inheritance cases sourced from official Saudi curriculum texts and reliable digital resources. The dataset contains fundamental concerns and related assessment questions designed to evaluate various degrees of model reasoning.

### 2.1 Existing datasets on Islamic inheritance reasoning

To assess Arabic language models on Islamic inheritance reasoning (ʿIlm al-Mawārīth/علم المواريث), the Question-and-Answer in Islamic Studies Assessment Shared Task (QIAS 2025) established a benchmark [8]. With its inheritance subtask, which emphasizes rule-based reasoning based on Islamic jurisprudence, models are evaluated using automatically generated multiple-choice questions (MCQs) categorized by degree of difficulty. In contrast, the MirathQA dataset was manually curated utilizing real-world examples from sources of classical jurisprudence as well as educational materials. The QIAS benchmark is complemented with a visible, comprehensible, and research-focused resource called QIAS 2025, which arranges questions according to reasoning tasks rather than difficulty levels. These tasks include computing shares, identifying the case base, and handling blocked heirs.

## 3 Data Description

The dataset excel file comprises:

• **Base Cases Sheet**: Over 240 annotated inheritance scenarios with the following fields: case id, description, answer, all heirs, share calculations, calculations, base, cases, comments and resource, details of each field are illustrated in Table 1. All cases in this dataset adhere to the Hanbali school of jurisprudence (المذهب الحنبلي), which is the authoritative madhab in Saudi Arabia and has specific rulings on inheritance shares.

**Table 1:** Data structure of the base cases sheet.

Field Name	Description
case_id	Unique identifier for each inheritance problem
description	Full textual description of the problem as in the source
answer	Full textual answer as in the source
all persons	List of heirs involved
share calculations	Allocated proportional share of the inheritance for each heir, with blocked heirs assigned a value of zero.
calculations	Portion for each heir relative to the base or as a fraction of the base
base	Denominator used to calculate final shares
cases	Type of special cases if any
resource	Direct link to original problem

• **Derived QA Sheets**: Multiple questions were derived from each scenario, in the form of five sets of multiple-choice questions and one set of true/false questions. These questions assess specific reasoning elements such as inheritance logic, heir eligibility, share calculation and special cases. A summary of these questions is presented in Table 2.

**Table 2:** Summary of derived QA sheets.

Category		Number of Questions	Questions Type	Answer's Data Type	Notes
A	Share-based reasoning (heirs' portions)	631	MCQ	Numeric + Nominal	Generated to test numeric reasoning over individual shares
B	Base fraction calculations	123	MCQ	Numeric	Focused on total base denominators and share normalization
C	Questions on blocking	146	MCQ	Nominal	Evaluates model understanding of blocked heirs (ḥajb)
D	Questions on blocking	242	T/F	Binary	Verifies binary understanding of heir eligibility
E	Special case scenarios	24	MCQ	Nominal	Special cases like ʿawl/عول, or unique inheritance logic
F	Total share distribution (Sahm/سهم)	228	MCQ	Numeric	Tests whether the model can deduce total number of shares used
	Total	1394

The released dataset is structured into three top-level directories:

• Train (70 % of cases for model development)
• Val (15 % of cases for hyperparameter tuning and model selection)
• Test (15 % of cases for final evaluation)

Each directory contains the same set of files, Mirath Dataset_final.csv (original inheritance cases) and the derivative question files (A–F), but restricted to the cases assigned to that partition. This ensures that all information associated with a particular case remains together in the same split.

For usability, two file formats are provided:

1. CSV files, saved with utf-8-sig encoding to ensure correct rendering of Arabic text in Microsoft Excel and compatibility with programming environments.
2. Excel (XLSX) workbooks, where each partition is provided as a workbook, and each file (Main, A–F) is represented as a separate sheet.

This design allows users to either process the dataset programmatically (via CSV) or explore it manually and intuitively (via Excel).

This organization ensures both machine-readability and human accessibility, while preserving the integrity of the splits and enabling reproducible experiments across different environments.

The dataset repository includes a comprehensive set of Python scripts that implement the data integrity verification, Mirath MCQs generation, and stratified data splitting and verification. These scripts enable researchers to confirm the dataset's accuracy (like checking for consistency among shared data, verifying blocked heirs, and ensuring calculations are correct) and to recreate all MCQ sheets (A-F) and splits.

## 4 Experimental Design, Materials and Methods

**Fig. 1:**
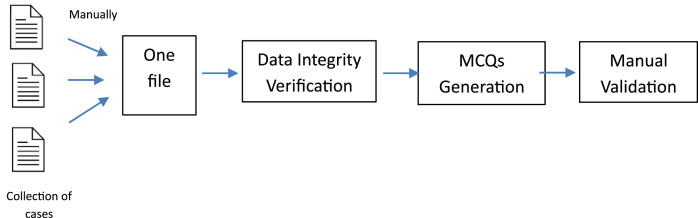
Study methodology.

This dataset does not involve human participants, surveys, questionnaires, or respondents. All data entries correspond to jurisprudential inheritance cases extracted from authoritative Fiqh sources. This study employs a systematic methodology for collecting and organizing information on Islamic inheritance law from credible and publicly accessible sources. Verified instructional materials, including the “Top 100 Inheritance Problems” collection [9], official Fiqh textbooks issued by the Saudi Ministry of Education [10], and other authoritative Islamic law websites [11], were utilized to ensure accuracy, consistency, and reliability of the data. All these sources are publicly available to use. Fig. 1 illustrates the detailed methodology followed in this study.

Since the dataset was populated manually, it is essential to verify its accuracy and ensure that no errors were introduced during data entry. The dataset comprises inheritance distribution cases, including details about the heirs and their allocated shares. As this dataset will serve as the foundation for automatic question generation and reasoning tasks, we implemented rigorous validation procedures through automated scripts developed in Python to systematically assess data consistency, accuracy, and correctness. The following section provides a detailed description of these validation steps.

### 4.1 Data integrity verification

We applied four computational validation methods to ensure each case was consistent and machine-ready: (1) normalization guidance via unique name frequencies, (2) persons–shares count matching, (3) duplicate case detection, and (4) strict share-format validation. These safeguards reduced entry errors, standardized entities, and removed redundancies before MCQ generation.

#### 4.1.1 Unique person name occurrence

This step generates a comprehensive table of all unique heir names in the dataset, along with the cases in which they appear, to identify spelling inconsistencies and orthographic variations. By identifying differences such as “أم/Mother” versus “الأم/The Mother,” it supports the manual unification of names and ensures consistency across the dataset. Additionally, maintaining a standardized list of names helps in generating random answer options for MCQs by selecting only from names that actually exist in the dataset for a given person, thereby avoiding the inclusion of irrelevant or obviously incorrect options that an LLM could easily recognize as wrong, which would otherwise undermine the proper evaluation of LLM performance.

Example:

• Found: {“Mother/أم” and “The Mother/الأم”} → unify to a single canonical form (e.g., “الأم”).
  Fix: Replace variants with the chosen canonical label across the dataset.

#### 4.1.2 Mismatch value count (Persons vs. shares)

This verification step ensures that each row in the dataset contains the same number of heirs and corresponding shares, helping to detect manual entry errors such as missing shares, extra shares, or forgotten commas that may cause misalignment between persons and their allocated shares. This check guarantees data completeness and consistency before proceeding to automated MCQ generation.

Example:

• Persons: ["أم/Mother","زوجة/Wife","إبن/Son"] → 3 items
• Shares: ["1/6","1/8"] → 2 items → flag (counts differ: 3 vs 2)
  Fix: Add the missing share or correct the persons list.

#### 4.1.3 Duplicate case detection

Since the data was collected by four different authors from multiple sources, there is a higher possibility of duplicate cases in the dataset. To address this, this step identifies and flags rows containing identical (exact match) lists of heirs and, optionally, matching share distributions. Removing these redundant cases ensures a clean, consistent dataset and prevents the repetition of scenarios that add no additional learning value.

Example:

• Detected Duplicate: Row 21 and Row 213 have identical lists of heirs → flagged as potential duplicate. ^‘^الزوج،الأم،الأب ^’^ → rows 21, 213
• Fix: Review the cases to confirm whether it is an actual duplication or a data entry error. If confirmed as a duplicate, retain one entry and remove or merge the redundant row.

#### 4.1.4 Share format validation

This step validates the format of all share values to ensure they strictly follow the correct representation: either as a fraction in the form number/number (e.g., 1/6, 2/3) or as a wildcard symbol * that means remaining shares. The verification process helps detect manual entry errors such as 1/6/ instead of 1/6, 1.4 instead of 1/4, or other malformed inputs. Any incorrect formats are flagged for manual review and correction to maintain data consistency and prevent parsing issues during automated processing.

Examples:

• 1/6/ → Invalid (trailing slash in Row 120)
• 1.4 → Invalid (decimal used instead of a fraction in Row 85)
• `` (empty value) or 1//4 → Invalid (double slash detected)

Fix: Review the flagged entries and correct them to valid formats, such as 1/4 or * for remaining shares.

### 4.2 MCQ generation methodology

To create a comprehensive and diverse question set, we developed a systematic, computational pipeline to automatically generate multiple-choice and true/false questions from the verified inheritance dataset. The methodology leverages the structure and rules of Islamic inheritance law to ensure that each question is contextually accurate, domain-specific, and pedagogically meaningful. Various question types were designed to evaluate LLMs understanding of shares, base numbers, blocked heirs, special inheritance scenarios, and share calculations.

#### 4.2.1 Shares of heirs

For each solved inheritance case, we generated one multiple-choice question (MCQ) per heir. For example, if a case involves six heirs, we create six separate MCQs, with each question focusing on the share of a specific heir. Every MCQ consists of six answer options: one correct answer representing the heir’s actual share and five distractors randomly sampled from the dataset using all known possible shares for that heir. For instance, when generating a question about “الأم/The Mother” the distractors are chosen exclusively from the documented shares of the mother in other cases. This approach ensures that all answer options are plausible, domain-specific, and contextually accurate rather than arbitrary, making the questions more challenging and realistic. Additionally, the process guarantees that no duplicate or identical distractors appear within the same question. The following code snippet illustrates the structure used for generating these questions.

“Question”: f" {row[description]} فكم نصيب {person}?”

#### 4.2.2 Base of the case

The Aṣl al-Masʾalah/أصل المسألة (base number) is the smallest integer from which all heirs’ shares are distributed without fractions. It is derived from the denominators of the prescribed shares, typically using the least common multiple (LCM), with common values being 2, 3, 4, 6, 8, 12, and 24 [12].

For each case with a verified base, we generate one MCQ asking: “What is the base denominator of the shares for this case?” Each MCQ provides six answer options: one correct answer representing the documented base and five randomly sampled from the bases of other cases in the dataset. The following code snippet demonstrates the question structure used to generate these base-related questions.

“Question”: f” ما هو أصل المسألة التالية {description}?”

#### 4.2.3 Blocked heirs MCQs

In Islamic inheritance, a blocked heir (ḥajb/حجب) is a rightful inheritor who receives no share because another heir with a stronger entitlement exists. We generate multiple-choice questions (MCQs) to evaluate understanding of blocking rules. Only cases where at least one heir is blocked and the total number of heirs is three or more are used, since fewer heirs would not provide enough options to form six plausible answers. Each MCQ asks “Who is blocked in this case?” and provides six answer choices: a mix of actual heirs from the case and plausible distractors, with exactly one correct answer representing the blocked heir(s). The following code snippet demonstrates the structure used for generating blocked-heir MCQs.

“Question”: f” من من الورثة لا يرث في المسألة التالية {description}?”

#### 4.2.4 Blocked heirs true/false

In addition to MCQs, we generated True/False (T/F) questions to test whether learners can identify the presence of blocked heirs. For every inheritance case, we created one T/F question asking “Is there any blocking in this case?”. The correct answer is True if at least one heir’s share equals 0 and False if no heirs are blocked. The following code snippet illustrates the structure used for generating these True/False questions.

“Question”: f” هل يوجد حجب من الورث في المسألة التالية {description}?”

#### 4.2.5 Special cases

Special inheritance scenarios arise when standard share distribution rules are modified due to exceptional conditions. Examples include: al-ʿUmariyyātān/العمريتان (mother receives ⅓ of the remainder), al-ʿAul/العول (proportional reduction when total shares exceed the estate), al-Mushārakah/المشاركة (maternal siblings sharing with full siblings), al-Radd (leftovers distributed to fixed-share heirs), and al-Munāsakhāt/المناسخات (successive inheritance when an heir dies before distribution). MCQs are generated only for cases explicitly marked as special in dataset references. The following code snippet shows the structure used for generating special-case questions.

“Question”: f” ماهي الحالة الصحيحة للمسألة التالية {description}?”

#### 4.2.6 Share calculation

This step generates MCQs about the numerical share values (integers) of heirs relative to the base number (Aṣl al-Masʾalah/أصل المسئلة). For each heir with a non-zero share, we create one MCQ asking “What is the number of shares allotted to [specific heir] from the base number of the case?”. Each question provides six answer options: one correct numerator and five distractors randomly sampled from numerators of the same heir in other cases, ensuring plausible alternatives. The following code snippet demonstrates the structure used for generating these share-calculation questions.

“Question”: f” إذا كان أصل المسألة { base} ماهو نصيب {person في المسألة التالية{description}?”

Through this structured pipeline, 1394 MCQs and T/F questions were generated, grounded in Islamic inheritance law and validated references. By leveraging plausible distractors and dataset-driven logic, the methodology ensures authentic, challenging, and pedagogically effective assessments suitable for both human learners and LLM evaluation. Table 3 shows some examples from each questions type.

**Table 3:** Examples from each question category.

Type	case_id	Question_id	Question	Choices	Answer	CorrectText
A	C008	A016	توفي شخص عن أبيه وابنه فكم نصيب الأب؟ A man died leaving his father and his son. What is the father's share of inheritance?	[A-2/3, B-1/6, C-1/2, D-5/6, E-2/5, F-1/3]	B	1/6
B	C078	B013	ما هو أصل المسألة للحالة التالية: توفي رجلعن زوجته وأخته لأبيه وعمه A man died leaving his wife, his paternal half-sister, and his paternal uncle. What is the root of the inheritance case?	[A-3, B-24, C-8, D-4, E-5, F-2]	D	4
C	C120	C032	من من الورثة لا يرث في المسألة التالية: توفيت امراه عن زوج واب وجد واخ وابن A woman died leaving her husband, father, grandfather, brother, and son. Who among them does not inherit?	[A-الأب, B-الزوج و الابن, C-الزوج, D-الأب و الابن, E-الابن, F-الأخ]	F	الأخ
D	C025	D025	هل يوجد حجب من الورث في المسألة التالية: توفي شخص عن أمه وجده A man died leaving his mother and his grandfather. Is there any exclusion (ḥajb) among the heirs?		F	
E	C019	E018	ماهي الحالة الصحيحة للمسألة التالية: توفي رجل عن زوجته وأمه وأبيه A man died leaving his wife, mother, and father. What is the correct distribution of inheritance?	[A-الدينارية, B-العمرية, C-الخرقاء, D-مختصرة زيد, E-الأخ المبارك, F-عول]	B	العمرية
F	C137	F051	إذا كان أصل المسألة 8 ما هو نصيب البنت في المسألة التالية: مات وترك: زوجـة وأب وبنت If the root of the inheritance case is 8, what is the daughter's share in the following case: A man died leaving a wife, a father, and a daughter?	[A-16, B-13, C-7, D-17, E-4, F-8]	E	4

### 4.3 Post manual validation

After generating all multiple-choice and true/false questions, we conducted a manual quality check on a random 10 % sample of the dataset to verify that the generated items were accurate, consistent, and faithfully aligned with the original dataset. During this review, each selected question was compared against the original inheritance case, verifying the correct answer, the plausibility of distractors, and adherence to Islamic inheritance rules. No inconsistencies were detected during this review, which further validates the robustness and reliability of the automated generation process.

### 4.4 Dataset partitioning

This section provides an overview of the dataset structure and its components. The dataset presented in this article comprises the primary inheritance cases file (Mirath Dataset_final.csv), which represents the core component of the inheritance dataset. Earlier versions included derivative files (Mirath Dataset_final_A.csv to F.csv) containing question formats such as multiple-choice and true/false; however, these files were discarded, as the final uploaded dataset already provides the processed and split versions (training, validation, and testing). Each entry in the dataset retains the case_id attribute to ensure that all generated questions remain linked to their corresponding inheritance scenarios.

**Fig. 2:**
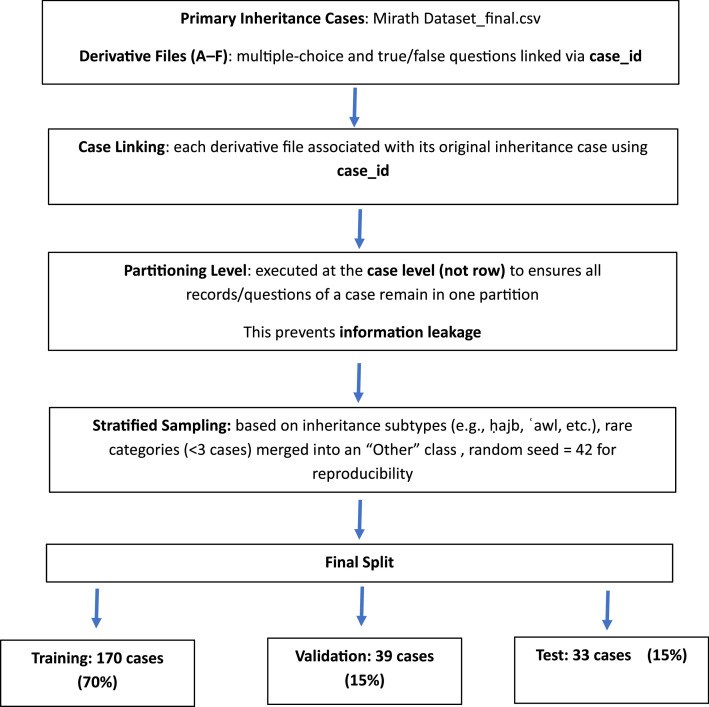
Inheritance dataset partitioning process.

The dataset was partitioned into three segments: training (70 %), validation (15 %), and test (15 %) to provide fair assessment and reproducibility. To prevent information leaking, the division was executed at the case level (utilizing the case_id field) rather than at the row level. This guarantees that all records pertaining to a certain inheritance case, including the initial case description and any associated derivative inquiries, are entirely housed within a single partition. The test set accurately simulates real-world evaluation conditions by incorporating entirely novel cases. Fig. 2 shows a flowchart of the Inheritance dataset partitioning process and how cases are spread out among the partitions.

To maintain representativeness across the partitions, a stratified sampling strategy was used. The stratification label was the cases column, which divides inheritance problems into subtypes (e.g., ḥajb [exclusion], ʿawl [proportional reduction], and other special categories). To guarantee balanced representation, rare categories (those with fewer than three cases) were merged into a generic “Other” class. Table 4 shows how cases are spread among stratums. The 70/15/15 ratio was used to randomly assign case identifiers within each stratum into train, validation, and test sets. To guarantee reproducibility, a fixed random seed (42) was used.

**Table 4:** Summary of how cases are spread among stratums.

stratum	train	validation	test
**None**	31	7	6
**Other**	12	3	2
**al-Akdariyyah/ الأكدرية**	2	1	0
**al-ʿUmariyya/ العمرية**	2	1	0
**Taʿṣīb/تعصيب**	67	14	15
**ḥijb/حجب**	31	7	6
**ḥijb,Taʿṣīb/تعصيب حجب،**	21	4	5
**radd/رد**	4	1	0

We validated the integrity of the train/validation/test partition through four checks:

1. **Case-level leakage**: we verified that each case_id appears in exactly one partition by computing set intersections of case_ids across splits; no overlaps were found.
2. **Ratio compliance**: we measured the proportion of unique cases in each split and confirmed the target 70 %/15 %/15 % ratio (within rounding tolerance).
3. **Stratification balance**: we compared frequency distributions of the cases category across splits to ensure that major inheritance subtypes (e.g., ʿ we, ḥajb) are represented in each partition; rare categories were merged into “Other” prior to splitting among splits.
4. **Consistency of derived files**: for all derived question files (A–F), we verified that their case_ids are a subset of the case_ids assigned to the corresponding split in the main file, guaranteeing that no derived item crosses partitions. All checks passed, confirming that the split is leakage-free, ratio-compliant, and stratification-consistent.

## Limitations

The dataset does not cover every rare or controversial scenarios in Islamic inheritance. Some cases reflect the dominant Sunni (Hanbali/حنبلي) interpretation and may not generalize across all schools of jurisprudential thought. All examples are in Arabic, requiring language-specific model capabilities.

## Ethics Statement

This work does not involve human subjects or respondent data, animal experiments, or social media data. All data points are drawn from public, educational, and legally published sources. The authors affirm compliance with Data in Brief’s ethical guidelines.

## CRediT authorship contribution statement

**Ameera Almasoud:** Conceptualization, Methodology, Software, Validation, Formal analysis, Investigation, Resources, Data curation, Writing – original draft, Writing – review & editing, Visualization. **Sharefah Al-Ghamdi:** Conceptualization, Methodology, Software, Validation, Formal analysis, Investigation, Resources, Data curation, Writing – original draft, Writing – review & editing, Visualization. **Reem Alqifari:** Conceptualization, Methodology, Software, Validation, Formal analysis, Investigation, Resources, Data curation, Writing – original draft, Writing – review & editing. **Noof Alfear:** Conceptualization, Methodology, Software, Validation, Formal analysis, Investigation, Resources, Data curation, Writing – review & editing. **Hend Al-Khalifa:** Conceptualization, Writing – review & editing.

## Data Availability

Mendeley DataMirathQA (Original data)

## References

[1] Siregar A. (2021). Inheritance distribution in Islam between legal provisions and dynamics of its implementation. Int. J. Res. Innov. Soc. Sci..

[2] Zuleika A. (2018). Islamic inheritance law (Faraid) and its economic implication. Int. J. Nusant. Islam.

[3] Yassari N., Reid K.G.C., de Waal M.J., Zimmermann R. (2020). Comparative Succession Law.

[4] Qur’an, Surah An-Nisa, Verse 11, Qur’an.com, https://quran.com/4/11 (accessed 19 September 2025)

[5] Qur’an, Surah An-Nisa, Verse 12, Qur’an.com, https://quran.com/4/12 (accessed 19 September 2025).

[6] Qur’an, Surah An-Nisa, Verse 176, Qur’an.com, https://quran.com/4/176 (accessed 19 September 2025

[7] Nguyen H.T., Sætra H.S., He J., Aras H. (2025). LLMs for legal reasoning: A unified framework and future directions. J. Comput. Sci..

[8] Bouchekif A., Rashwani S., Mohamed E.S.A., Alkhatib M., Sbahi H., Gaben S., Zaghouani W., Erbad A., Ghaly M. (2025). Proceedings of the Third Arabic Natural Language Processing Conference: Shared Tasks, Association for Computational Linguistics.

[9] AlMwareeth Website, Islamic inheritance cases and resources, https://almwareeth.com (accessed 12 September 2025).

[10] Saudi Ministry of Education, Fiqh Textbook, Secondary School, Level 3, Ministry of Education, Riyadh, Saudi Arabia, 2022 (in Arabic).

[11] Shamela Digital Library, Islamic texts and resources, https://shamela.ws (accessed 12 September 2025).

[12] Ibn Qudamah, Al-Mughni, Dar Alam Al-Kutub, Riyadh, Saudi Arabia, 1997.

